# More natural more better: triple natural anti-oxidant puerarin/ferulic acid/polydopamine incorporated hydrogel for wound healing

**DOI:** 10.1186/s12951-021-00973-7

**Published:** 2021-08-11

**Authors:** Qianmin Ou, Shaohan Zhang, Chuanqiang Fu, Le Yu, Peikun Xin, Zhipeng Gu, Zeyuan Cao, Jun Wu, Yan Wang

**Affiliations:** 1 Hospital of Stomatology, Guanghua School of Stomatology, Sun Yat-sen University, Guangdong Provincial Key Laboratory of Stomatology, Guangzhou, 510055 China; 2 School of Biomedical Engineering, Sun Yat-Sen University, Shenzhen, 518107 China; 3 College of Polymer Science and Engineering, State Key Laboratory of Polymer Materials Engineering, Sichuan University, Chengdu, 610065 China

**Keywords:** Puerarin, Ferulic acid, Polydopamine, Hydrogel, Wound healing

## Abstract

**Background:**

During wound healing, the overproduction of reactive oxygen species (ROS) can break the cellular oxidant/antioxidant balance, which prolongs healing. The wound dressings targeting the mitigation of ROS will be of great advantages for the wound healing. puerarin (PUE) and ferulic acid (FA) are natural compounds derived from herbs that exhibit multiple pharmacological activities, such as antioxidant and anti-inflammatory effects. Polydopamine (PDA) is made from natural dopamine and shows excellent antioxidant function. Therefore, the combination of natural antioxidants into hydrogel dressing is a promising therapy for wound healing.

**Results:**

Hydrogel wound dressings have been developed by incorporating PUE or FA via PDA nanoparticles (NPs) into polyethylene glycol diacrylate (PEG-DA) hydrogel.

This hydrogel can load natural antioxidant drugs and retain the drug in the gel network for a long period due to the presence of PDA NPs. Under oxidative stress, this hydrogel can improve the activity of superoxide dismutase and glutathione peroxidase and reduce the levels of ROS and malondialdehyde, thus preventing oxidative damage to cells, and then promoting wound healing, tissue regeneration, and collagen accumulation.

**Conclusion:**

Overall, this triple antioxidant hydrogel accelerates wound healing by alleviating oxidative injury. Our study thus provides a new way about co-delivery of multiple antioxidant natural molecules from herbs via antioxidant nanoparticles for wound healing and skin regeneration.

**Graphic Abstract:**

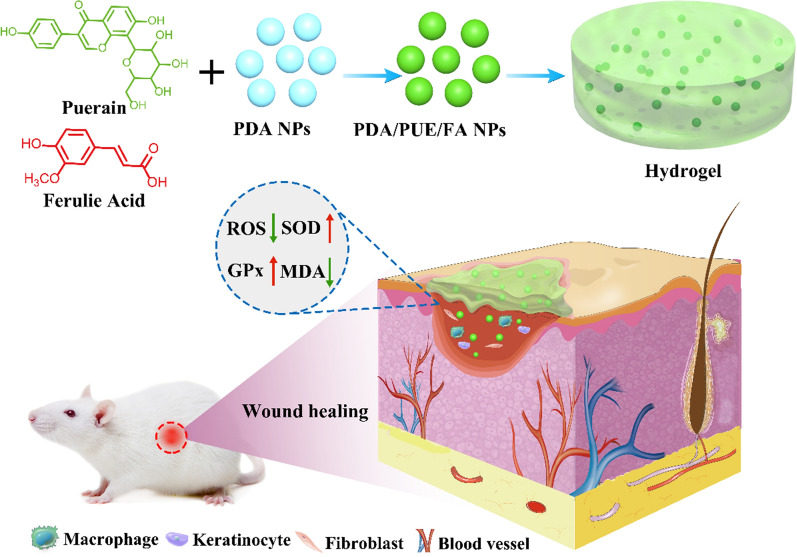

**Supplementary Information:**

The online version contains supplementary material available at 10.1186/s12951-021-00973-7.

## Introduction

Wounds are common injuries that can lead to skin breakage or opening, which can cause acute and chronic infections [1, 2]. Reactive oxygen species (ROS), including superoxide radicals and non-radical hydrogen peroxide, are harmful to wound healing because of their detrimental effects on tissues and cells [3]. Drugs with free radical-scavenging properties have been proven to significantly promote wound healing against oxidative damage following topical administration to patients [4].

Biomaterial-based drug delivery systems capable of eliminating these harmful ROS may facilitate therapeutic effects on wound healing. In recent years, hydrogels as a biomaterial show the advantages in many ways for their good biodegradability and biocompatibility. In addition to their physiochemistry similar to the native extracellular matrix, which can act as supporting material for drug-controlled release system.

Nowadays, there are many different types of nanostructured drug delivery systems for wound healing, such as micelles, liposomes, inorganic nanoparticles and polymeric nanoparticles (Additional file 1: Table S1). PDA nanoparticles (NPs), as a drug carrier, possess sustained drug release ability and excellent antioxidant activity due to the nanosized effects [5–7]. Additionally, the amine groups of PDA can help chemicals feasibly conjugate on the surface of PDA NPs [8]. Therefore, PDA NPs is an ideal drug carrier for drug delivery in hydrogel.

Puerarin (PUE), a natural flavonoid, demonstrates various pharmacological activities, such as anti-inflammatory and antibacterial effects [9]. Furthermore, PUE can inhibit lipid peroxidation by reducing superoxide anion production [10, 11]. Ferulic acid (FA) is a phenolic compound, which is known for its excellent antioxidant activity [12]. FA exerts therapeutic effects on various diseases, including cardiovascular disease, cancer, and skin disease, owing to its free radical-scavenging ability [12, 13].

**Fig. 1:**
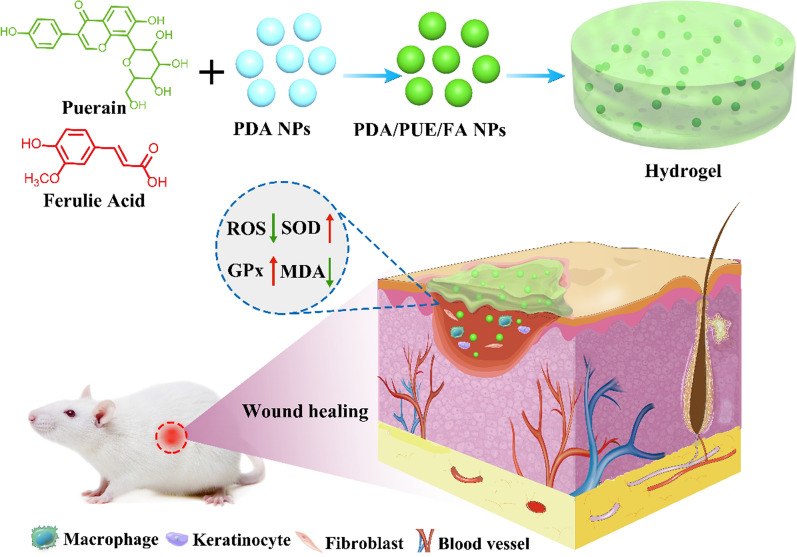
Schematic showing the development of hydrogels for wound healing applications. The hydrogel (PEG-DA/PDA/PUE/FA hydrogel) protects cells from the damage from oxidative stress and promotes the formation of collagen fibers and blood vessels, which accelerates wound healing

However, the challenge for wound dressings is to alleviate oxidative stress effectively. It is worth to test whether the integration of multiple natural anti-oxidant drugs have maximized antioxidant function for wound healing. In present study, PUE and FA were incorporated into PDA NPs, then into a polyethylene glycol diacrylate (PEG-DA) hydrogel to form a three-dimensional PEG-DA/PDA/PUE/FA hydrogel network. PUE and FA were retained in this gel network for a long period because of the presence of PDA NPs, which was beneficial for wound healing (Fig. 1).

## Materials and methods

### Materials

For hydrogel fabrication, PEG-DA (MW = 8 kDa), dopamine, PUE, FA, and photoinitiator 2959 were purchased from Sigma-Aldrich. Human periodontal ligament stem cells (hPDLSCs) used herein were acquired from Guanghua School of Stomatology. Dulbecco’s Modified Eagle Medium (DMEM) and fetal bovine serum (FBS) were obtained from Gibco-BRL. Penicillin/streptomycin solution was purchased from HyClone. In cell proliferation test, colorimetric 3-(4,5-dimethylthiazol-2-yl)-5-(3-carboxymethoxyphenyl)-2-(4-sulfophenyl)-2H-tetrazolium) (MTS) assay kit was procured from Promega. Live/Dead Cell Double Staining Kit and 2,'7'-dichlorofluorescein diacetate (DCFH-DA) fluorescent dyes were purchased from Sigma-Aldrich. Superoxide dismutase (SOD), glutathione peroxidase (GPx), and malondialdehyde (MDA) test kits were obtained from Nanjing Jiancheng Biotechnology Institute. CD34 and VEGF antibodies were purchased form Servicebio. Sprague–Dawley rats were provided by the Laboratory Animal Center of Sun Yat-sen University. All animal surgeries were performed according to the guidelines of the Ethics Committee of Sun Yat-sen University.

### Drug-loaded PDA NPs

PDA NPs were synthesized using a previously reported method [14]. An aqueous solution of ammonia (25%, 0.5 mL) was added to a mixture of ethanol (4 mL) and deionized water (9 mL), and the resulting mixture was placed in a water bath at 40 ℃ under mild magnetic stirring. Dopamine hydrochloride (50 mg) was dissolved in deionized water (1 mL) and the product was obtained via centrifugation. The final product was suspended, and its concentration was determined by weighing after lyophilization. PUE (20 mg) and FA (20 mg) were mixed with PDA NPs (9 mL, 1.0 mg/mL) suspended in a mixture of water and ethanol (9:1, v/v). The mixed solution was incubated for 4 h, followed by centrifugation to precipitate drug-loaded PDA NPs (PDA/PUE, PDA/FA, and PDA/PUE/FA NPs). The size and morphology of drug-loaded PDA NPs were analyzed by dynamic light scattering and transmission electron microscope (TEM). Drug loading ratio was calculated according to the following formula: drug loading ratio = drug mass × 100%/(drug mass + PDA NP mass).

### Preparation of the hydrogel

PEG-DA powder (150 mg), different amounts of drug-loaded PDA NPs, and photoinitiator 2959 were mixed in deionized water. After vigorous stirring, the obtained mixture was transferred into a mold. PEG-DA, PEG-DA/PDA, PEG-DA/PDA/FA, PEG-DA/PDA/PUE, and PEG-DA/PDA/FA/PUE hydrogels were obtained by UV crosslinking for 300 s. To characterize the morphology of the hydrogels, they were swollen in water and freeze-dried using a freeze drier. Then, the samples were examined using a scanning electron microscope.

### Characterization of hydrogels

For determining the swelling kinetics, dry hydrogels were precisely weighed and submerged in the buffer solutions. Then the swelling ratio of the composite hydrogels was measured by gravimetric method using buffer solutions (pH: 7.4) at room temperature [15].

The degradation of hydrogels was gravimetrically monitored by carrying out hydrolytic degradation tests in the buffer solutions with shaking at 100 rpm [15]. At the predetermined time point, sample were taken out from solutions, dried in oven and weighted. The Remnant Weight (%) is W_t_/W_0_ × 100%. Where W_t_ and W_0_ are the dry weight of the sample after degradation at the time point and the dry weight of the initial sample, respectively.

For investigating the mechanical properties of the hydrogels, compression tests were conducted using an INSTRON tensile strength tester with 10 N at a crosshead speed of 10 mm/min. The water vapor transmission rate (WVTR) was measured using the ASTM E96-95 standard. The weight loss of the standard was calculated to measure the transfer of water vapors [16]. All measurements were performed three times.

### Antioxidant capacity of hydrogels

Antioxidant capacity of the hydrogels was assessed by monitoring their ability to eliminate 2,2-diphenyl-1-picrylhydrazyl (DPPH^•^) and hydroxyl radicals (^•^OH) [17]. For the DPPH^•^ assay, the liquid hydrogel precursor was added to the DPPH^•^ solution in methanol. The absorbance of the samples and blank was measured at 517 nm. Percentage of DPPH^•^ scavenging = (Ab—As) / Ab × 100%, where Ab and As are the absorbance of the blank and sample at 517 nm, respectively.

For the ^•^OH-scavenging assay, ^•^OH is generated from Fenton reaction between ferrous ions and hydrogen peroxide (H_2_O_2_). The reaction mixture, containing 1 ml sample solution, 1 ml salicylic acid (9 mM), 1 ml FeSO_4_ (9 mM) and 1 ml H_2_O_2_ (6 wt%), was incubated for 10 min, and then were heated at 37 °C for 30 min. Finally, the absorbance was recorded at 510 nm. The ^•^OH-scavenging ability of the liquid hydrogel precursor = (Ab—As)/Ab × 100%, where Ab and As are the absorbance of the blank and sample at 510 nm, respectively.

### In vitro drug release studies

In vitro release of PUE and FA from the hydrogel was carried out at 37 ℃ at a rotation speed of 100 rpm in 100 mL buffer. Then, the dry sample loaded with the drug was immersed in a buffer solution with the same composition. In a few time intervals, 5 mL solution containing the released drug was withdrawn, and 5 mL fresh solution was added to keep the solution volume constant. The drug concentration in the extracted solution was analyzed using a UV–Vis spectrophotometer, and the calibration curve was constructed using a series of PUE or FA solutions with known concentrations. All release experiments were performed in triplicate, and the average values were considered.

### Biocompatibility of hydrogels

Human periodontal ligament stem cells (hPDLSCs) were obtained as previously reported [18]. Passage 3–5 (P3-5) cells were used in related experiments. The cells were cultured in DMEM with 10% FBS and 1% penicillin/streptomycin at 37 ℃ and 5% CO_2_. Cellular viability was assessed by MTS assay. For extracting the liquids of hydrogels, 5 mg/mL hydrogels were added to DMEM under magnetic stirring at 37 ℃ for 24 h. The resulting solutions were passed through a 0.22 μm filter before being co-incubated with cells in a 96-well plate for 1, 4, and 7 days. After the MTS solution was processed, the absorbance at 490 nm was determined using an automatic microplate reader (BioTek, Winooski, VT, USA). For live/dead cell staining, cells were seeded onto a 48-well plate coated with hydrogels and incubated at 37 ℃. Subsequently, the cells were first stained with 2.5 μg/mL propidium iodide (PI) for 5 min, followed by 0.2 μg/mL calcein-acetoxymethyl ester (Calcein-AM) staining for 15 min. The cells were observed via fluorescence microscopy.

### Measurement of oxidative stress

ROS level was measured by DCFH-DA reagents using a flow cytometer. Briefly, hPDLSCs were seeded onto a 6-well plate coated with hydrogels for 24 h. H_2_O_2_ (100 μM) was added to stimulate oxidative stress for 24 h. Then, the cells were harvested, stained with DCFH-DA (6 μM) for 30 min, and analyzed using a flow cytometer. DCF% is F_s_ / F_C_ × 100% F_s_ is the florescence of sample group, and F_C_ is the florescence of control group.

Moreover, the levels of oxidative stress indicators were measured using superoxide dismutase (SOD), glutathione peroxidase (GPx), and malondialdehyde (MDA) test kits following the manufacturer’s protocols.

### In vivo wound healing

The effects of hydrogels on wound healing were evaluated in a rat model. Herein, Sprague–Dawley rats (body weight 250–300 g) were used. After administering pentobarbital (2%, 0.2 ml/100 g) anesthesia, the rat’s dorsal side was completely depilated, and a full-thickness circular wound (diameter: 20 mm) was created on the upper back of the mice. A blank wound without hydrogel was used as a control. The experiment was conducted in accordance with the protocol approved by the Institutional Animal Care and Use Committee of Sun Yat-sen University. After 15 days of healing, the entire wound including the surrounding normal skin was excised and fixed in 4% buffered paraformaldehyde. Then, the samples were embedded in paraffin, and 5-μm-thick sections were stained with hematoxylin and eosin (H&E) and Masson’s trichrome for histological analysis. Immunohistochemical staining was performed using antibodies against CD34 and VEGF.

### Statistical analysis

Statistical analysis was conducted using SPSS Statistics 20.0 software (IBM, Armonk, NY, USA). All data are expressed as the mean ± SD. One-way analysis of variance with Tukey’s test was used for comparison among groups. *P* < 0.05 was considered statistically significant.

## Results and discussion

### Preparation of PEG-DA/PDA/PUE/FA hydrogel

**Fig. 2:**
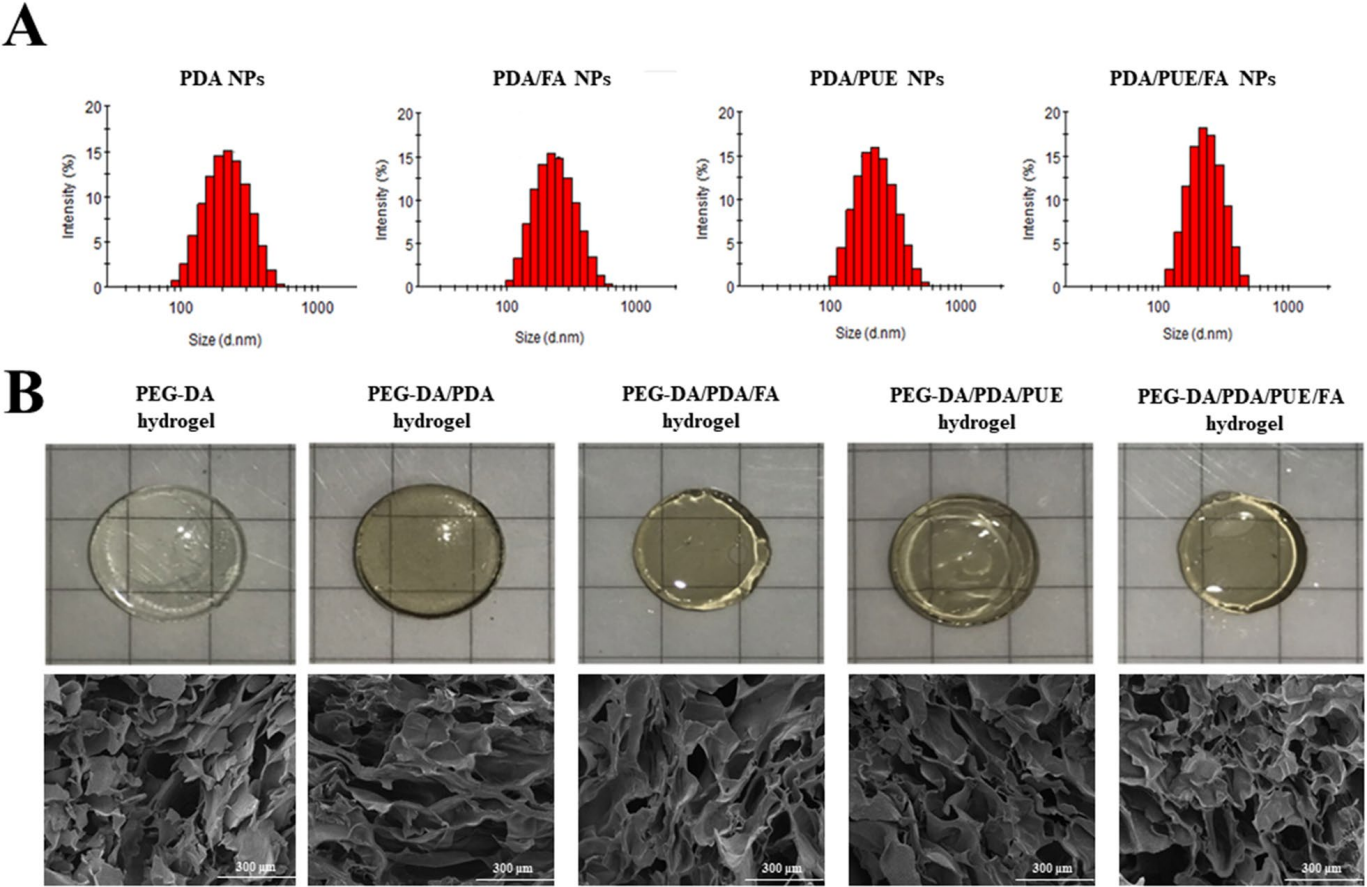
Diameters of NPs and morphology of hydrogels. Dynamic light scattering of PDA NPs, PDA/PUE NPs, PDA/FA NPs, and PDA/PUE/FA NPs (**A**). Images of the prepared hydrogels (**B**, upper lane, Scale bar is 1 cm). Scanning electron microscopy images of the hydrogels (**B**; lower lane)

Herein, PDA NPs, PDA/FA NPs, PDA/PUE NPs, and PDA/PUE/FA NPs were prepared. The size of the NPs was characterized by dynamic light scattering (Fig. 2A; PDA NPs: 193.28 ± 3.16 nm, PDA/FA NPs: 216.61 ± 5.19 nm, PDA/PUE NPs: 206.55 ± 3.81 nm, and PDA/PUE/FA NPs: 233.14 ± 4.67 nm). Similarly, TEM results showed that the average of PDA NPs, PDA/FA NPs, PDA/PUE NPs, and PDA/PUE/FA NPs were near 150–200 nm (Additional file 1: Fig S2). The diameter of PDA NPs is more than 100 nm, which may relate to the synthesis process of dopamine polymerization [19].

The FA loading ratios of PDA/FA NPs and PDA/PUE/FA NPs were 9.45 ± 0.89% and 6.09 ± 2.31%, respectively. The PUE loading ratios of PDA/PUE NPs and PDA/PUE/FA NPs were 7.93 ± 1.77% and 5.67 ± 1.89%, respectively. The drug loading ratio in PDA NPs is associated with the type of drugs and the proportion between drugs and NPs [20, 21]. The FT-IR spectrum of PEG-DA showed that two peaks at 1116 cm-1 and 1718 cm-1 are the ester group absorption peak, which indicated the existence of PEG-DA (Additional file 1: Fig S3). The peak at 1620 cm-1 is the stretching vibration of aromatic amine in PDA. The peak of 1632 cm-1 is the ketone group of PUE, and the peak of 1692 cm-1 is the stretching vibration of C = O of FA (Additional file 1: Fig S3).

Hydrogels were prepared by saturating the double bonds of PEG-DA via UV irradiation, resulting in the formation of a three-dimensional network structure (Fig. 2B). Although the pore size was not uniform, the three-dimensional structure of the hydrogel and small pores were distinctly observed in the dried hydrogel frame (Fig. 2B).

Several characteristics, such as properties and applications, especially biomedical applications, of a hydrogel depend on the pore size of the inner structure, dispersion of NPs, and morphology [22, 23]. For instance, the adsorption ability of a hydrogel is determined by the pore size, and the size of the drug determines whether the drug can be entrapped in the hydrogel network [24, 25]. Scanning electron microscopy results indicated that the PEG-DA/PDA/PUE/FA hydrogel was highly interconnected and infiltrated throughout the pores. It is speculated that the PEG-DA/PDA/PUE/FA hydrogel not only possesses high nutrient permeability but also improves cellular growth.

### Characterization of the PEG-DA/PDA/PUE/FA hydrogel

**Fig. 3:**
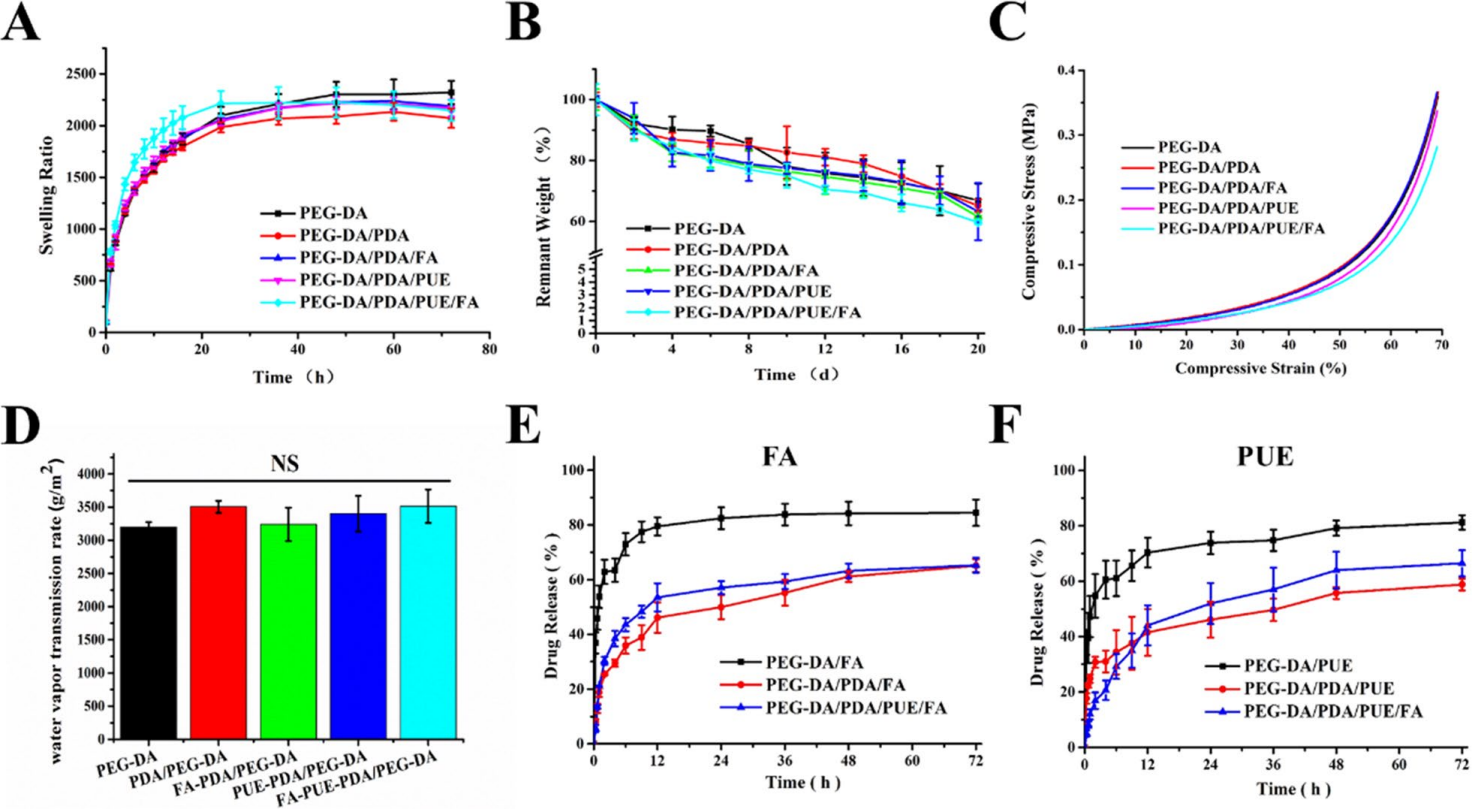
Characterization of the PEG-DA/PDA/PUE/FA hydrogel. Swelling behaviors (**A**), degradation behaviors (**B**), compressive properties (**C**), water vapor transmission rate (**D**), in vitro FA release profiles (**E**), and in vitro PUE release profiles of the hydrogels (**F**). NS: no significance

The swelling property of hydrogels was analyzed as a function of time (Fig. 3A). All the investigated hydrogels exhibited absorption behavior. The hydrogels absorbed approximately 2200 times more water than their own weight in nearly 50 h. In chronic wounds, excess exudates lead to bacterial growth around the injury, which causes microbial infection and delays healing [26]. Therefore, owing to their huge physical dimensions, these hydrogels can absorb a high volume of fluid, which facilitates wound healing [26, 27].

In the degradation test, the different hydrogels exhibited similar degradability mainly because the structure and quality of the gel components were not markedly different (Fig. 3B). Additionally, the size of the hydrogel samples stably decreased, which may be mediated through surface and internal erosion [28].

Compressive strength was investigated to study the mechanical properties of the hydrogels. Young’s moduli of the different hydrogels were 2.17 ± 0.80, 2.02 ± 0.86, 1.93 ± 0.60, 1.83 ± 0.69, and 1.45 ± 0.63 MPa (Fig. 3C). There were no significant differences among the groups. Studies report that the application dopamine reduces the stiffness and strength of hydrogel, which may relate to the hydrogel substrate, composition, and pore structures [29, 30]. Moreover, the mechanical properties of the synthesized hydrogels were in the range of those of biological tissues, such as the skin and articular cartilage [31], which is beneficial for biocompatibility.

Next, the WVTR was tested to evaluate the porosity of the hydrogels. The WVTR values of the different hydrogels were 3198.23 ± 76.19, 3506.19 ± 89.83, 3241.38 ± 250.90, 3402.00 ± 268.20, and 3515.18 ± 252.62 g/m^2^ (Fig. 3D). The water content and porosity of a dressing are closely related to wound healing. During wound healing, the water content of the skin increases, and a dressing with high WVTR can promote wound closure [16].

Drug release behavior of the drug-loaded hydrogels was examined. Although PEG-DA/PDA/PUE/FA hydrogel had an irregular porous structure, they could still diffuse from the hydrogel (Fig. 3E and F). An initial burst release of the drug was observed (within 12 h), followed by a steady release, which may be due to the high concentration gradient between the drug release media during the initial phase and a low concentration gradient during the subsequent phase. However, without PDA, the drug in the PEG-DA hydrogel was released considerably faster than the cases of other hydrogels, which indicated the stability of the PDA NPs. This preliminary discovery provides a potential method for preparing PEG-DA hydrogels with slow drug-release properties. In addition, ultrasonication can be used to promote the stability and precision of drug delivery system [32, 33], which may enhance the effect of PDA NPs on wound healing.

### Biocompatibility of the PEG-DA/PDA/PUE/FA hydrogel

**Fig. 4:**
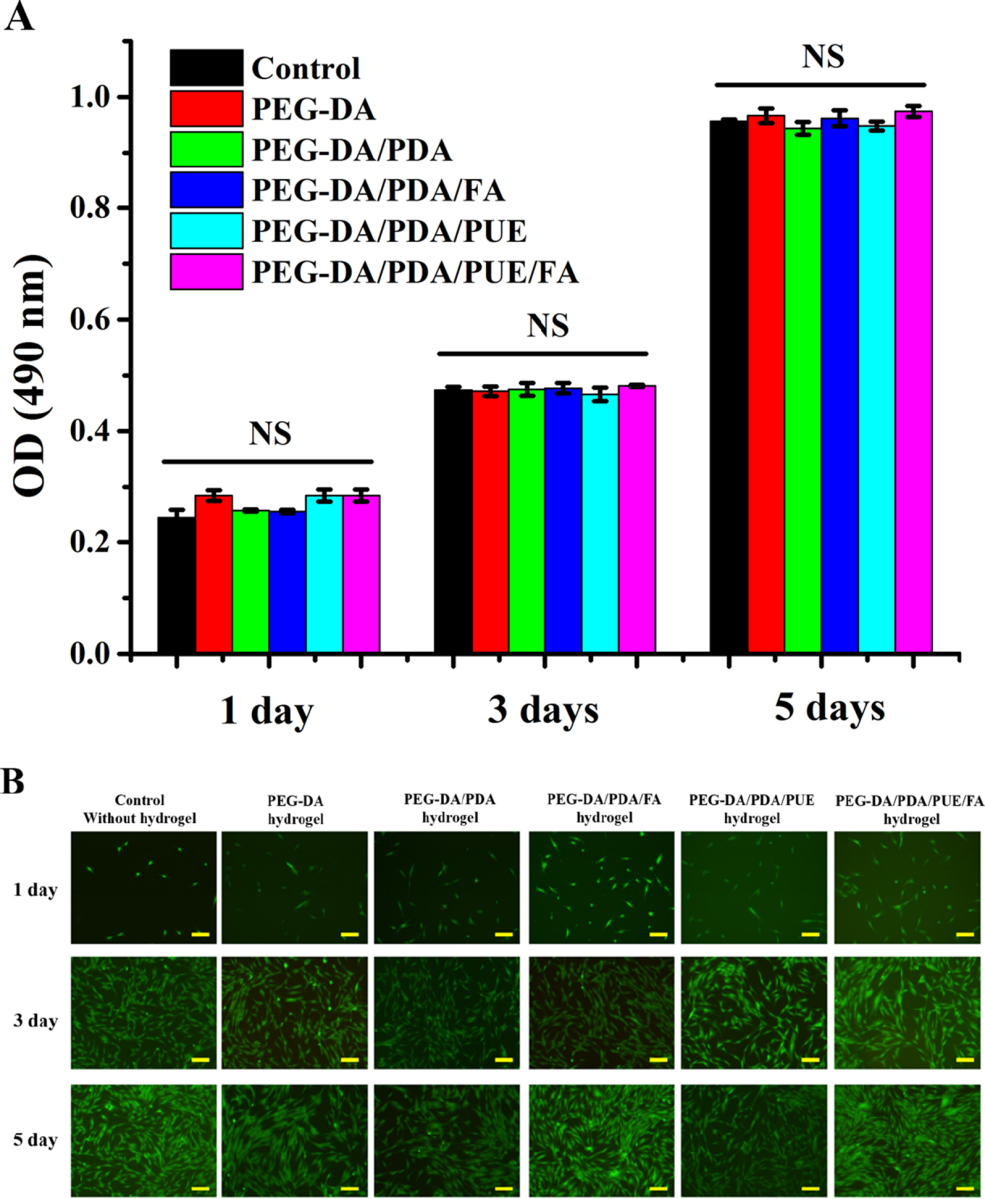
Biocompatibility of hydrogels. Proliferation of the cells cultured with the extracted liquids of hydrogels on different days (**A**). Calcein-AM/PI staining on different days after the cells were co-cultured with hydrogels (**B**; Scale bar is 100 μm). NS: no significance

In order to evaluate the biocompatibility of the hydrogels, hPDLSCs were isolated and characterized, which are accorded with the identification of mesenchymal stem cells (Additional file 1: Fig S1). Cells were co-incubated with the extracted liquids of the hydrogels. Cell proliferation was continuously detected over a period of 5 days (Fig. 4A). Under all conditions, the viability of the cells was maintained at a high level throughout the experiment, and the cells were capable of spreading and proliferating over time, indicating that the hydrogels did not have a marked detrimental effect on the long-term viability of hPDLSCs (Fig. 4A). Moreover, Calcein-AM and PI were used to monitor live and dead cells (Fig. 4B). These data verify that the different hydrogels proposed herein are conducive to cell growth and viability (Fig. 4B). These results further demonstrate the feasibility of using drug-loaded PDA NP-incorporated hydrogels with excellent biocompatibility for tissue engineering applications.

### Antioxidant activity and oxidative stress resistance ability of the PEG-DA/PDA/PUE/FA hydrogel

**Fig. 5:**
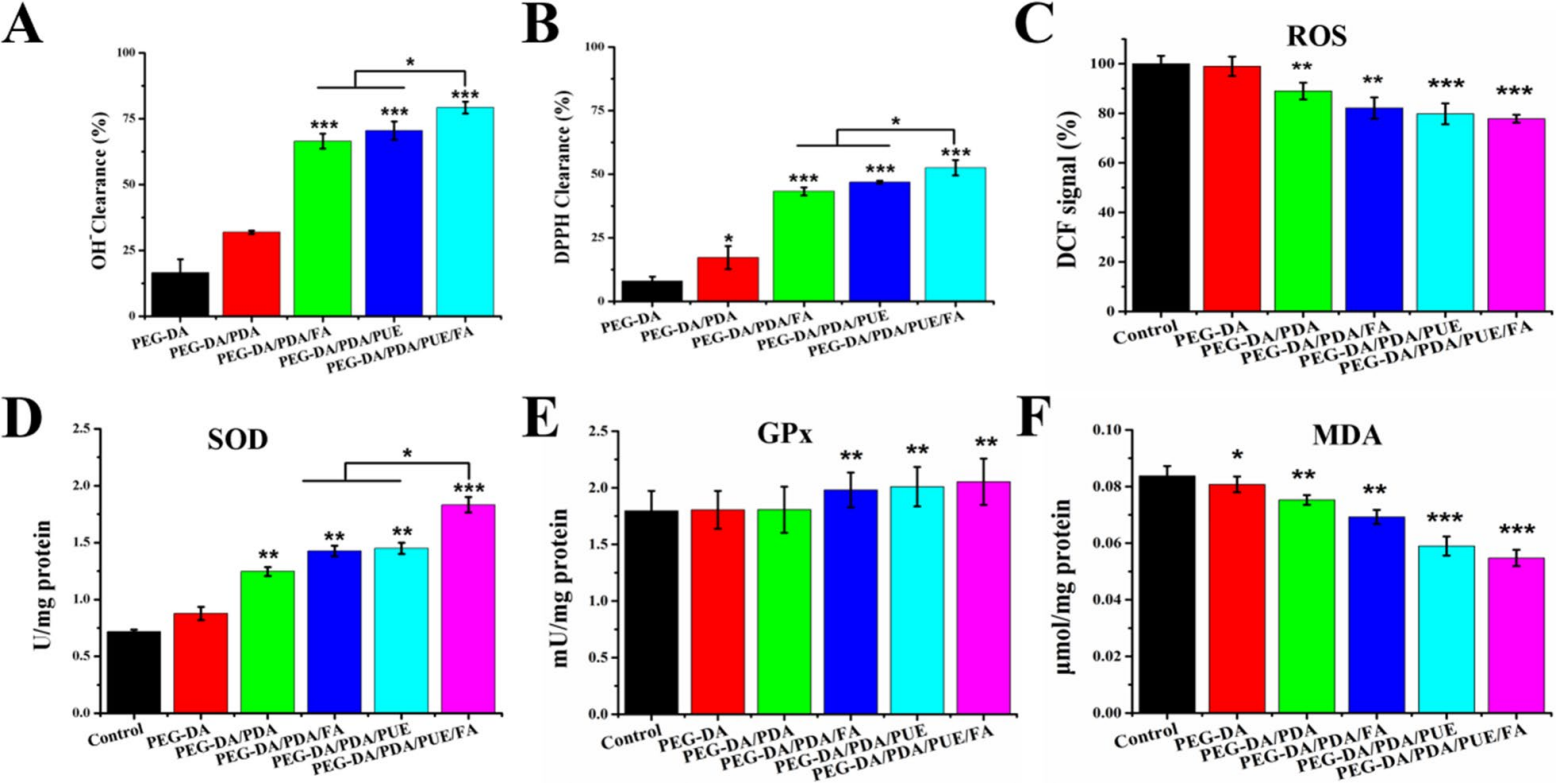
Antioxidant activity of hydrogels. Hydroxyl radical- (**A**) and DPPH^•^-scavenging ability of hydrogels (**B**). Changes in the ROS, SOD, and GPx levels and MDA content (**C**–**F**). ^*^P < 0.05, ^**^P < 0.01, and ^***^P < 0.001 when compared with the control group

Antioxidant activity of hydrogels is the key to inhibiting oxidative stress in tissue engineering. The antioxidant activity is measured by the DPPH^•^- and ^•^OH-scavenging ability of hydrogels and quantified as a percentage of the suppression of free radical formation [34, 35]. In the ^•^OH-scavenging assay, the scavenging of ^•^OH significantly improved after PEG-DA/PDA/PUE/FA hydrogel treatment (Fig. 5A). In the DPPH^•^ test, the elimination rate of DPPH^•^ also distinctly increased in the case of the PEG-DA/PDA/PUE/FA hydrogel group (Fig. 5B). More specifically, the PEG-DA/PDA/PUE/FA hydrogel demonstrated excellent antioxidant activity, with the maximum ^•^OH- and DPPH^•^-scavenging rates of 79.27 ± 2.20 and 52.55 ± 2.98% (Fig. 5A and B), respectively.

To investigate the oxidative stress resistance ability of the PEG-DA/PDA/PUE/FA hydrogels in embedded cells, we used H_2_O_2_ (100 μM) to directly expose the cells to superoxide radicals. The results showed that the introduction of the drug effectively suppressed the generation of intracellular ROS, and the fluorescence intensity of 2′, 7′-dichlorofluorescein in the PEG-DA/PDA/PUE/FA hydrogel was distinctly lower than that in the other hydrogels (Fig. 5C). Owing to the overproduction of ROS, cell biomolecules experience severe oxidative damage, causing disruption of the pro-oxidant-antioxidant balance [36]. SOD and GPx play a cytoprotective role under oxidative stress. Studies have shown that SOD is important for the oxidant and antioxidant balance in the body because it can eliminate superoxide anion free radicals as well as heal injured cells. GPx maintains the integrity of the cell membrane structure and is widely distributed in cells. Additionally, MDA produced by lipid oxidation can reflect oxidative stress injury caused by ROS. The PEG-DA/PDA/PUE/FA hydrogel effectively promoted the generation of SOD and GPx and inhibited the production of MDA (Fig. 5D–F). Therefore, the PEG-DA/PDA/PUE/FA hydrogel could protect the cells from oxidative stress damage.

Some reports demonstrate that PDA NPs can show some induced antioxidant activity due to the enriched phenol groups of PDA and the nanosize effects of NPs [7, 37]. In our study, we also found that, comparing with PEG-DA hydrogel, PEG-DA/PDA hydrogel could effectively increase the DPPH^•^ clearance and decrease ROS level. Moreover, PDA NPs can be used in periodontitis therapy due to the antioxidant capability [38].

PUE can react with free radicals because of its abundant surface electrons. It has been found to decrease Schwann cell apoptosis in a diabetic animal model owing to its antioxidant activity [39]. Furthermore, PUE significantly alleviates H_2_O_2_-induced oxidative stress injury and suppresses the apoptosis of neural cells [40]. FA has antioxidative, anti-inflammatory, and anti-hyperlipidemic properties [41]. The administration of FA reduces oxidative stress and DNA damage caused by lead acetate [42]. In our previous study, we showed that the hydrogel incorporated with PUE exerted excellent antioxidant effects, which promoted the regeneration and healing of damaged skin [43]. In the present study, we found that PUE and FA have a synergistic role in resisting oxidative stress damage in *vitro* and promoting wound healing in *vivo*. The PEG-DA/PDA/PUE/FA hydrogel also decreases cell death and enhances the survival capacity of hPDLSCs in an oxidative stress microenvironment, which is beneficial for wound healing.

### In vivo wound healing effects of the PEG-DA/PDA/PUE/FA hydrogel

**Fig. 6:**
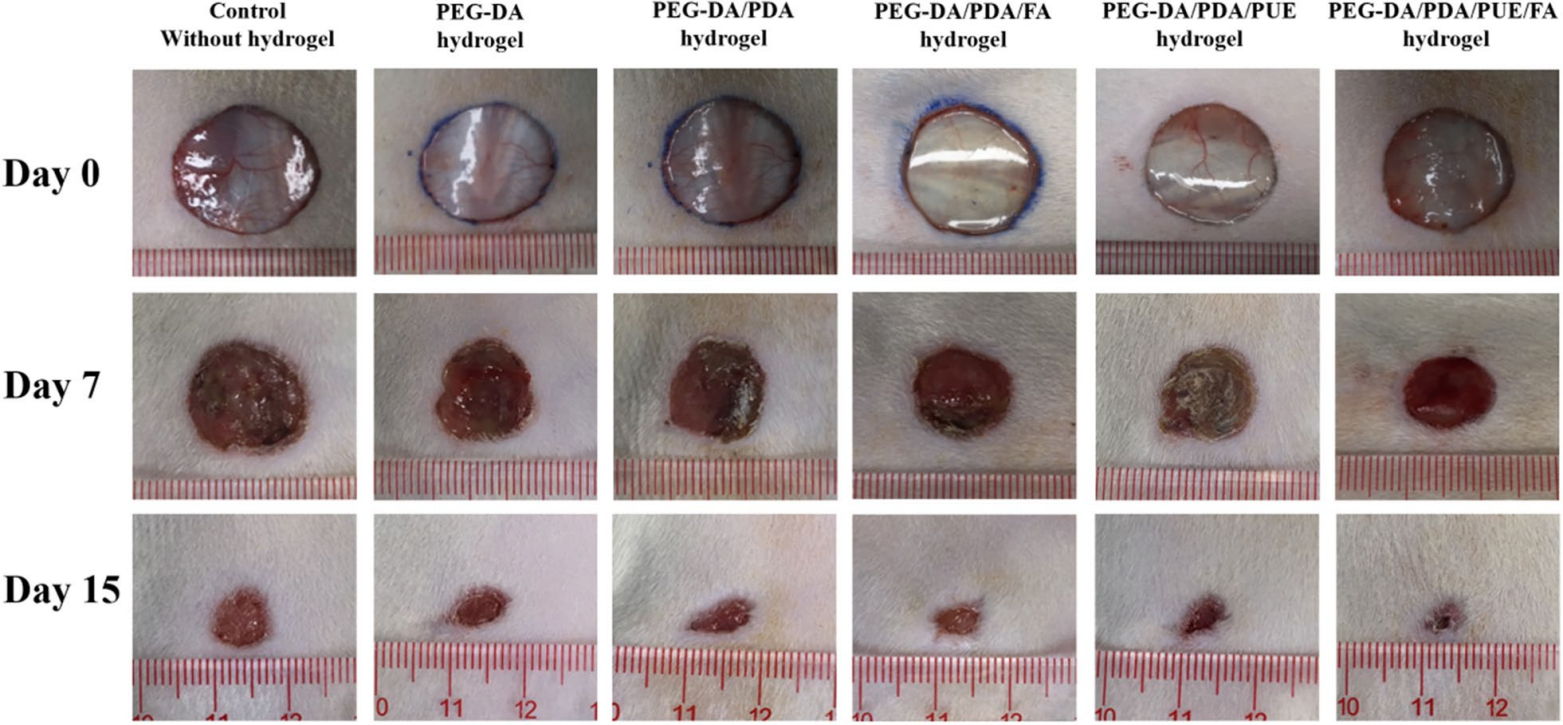
Wound healing effects of hydrogels. Macroscopic appearances of the skin wounds treated with hydrogels on days 0, 7, and 15

The wound healing properties of the hydrogels were further investigated by in vivo tests. The results demonstrated that the wounds treated with the PEG-DA/PDA/PUE/FA hydrogel healed faster than those treated with the other hydrogels (Fig. 6). On day 15, the wounds treated with the PEG-DA/PDA/PUE/FA hydrogel almost completely healed, whereas those treated with the control did not heal.

**Fig. 7:**
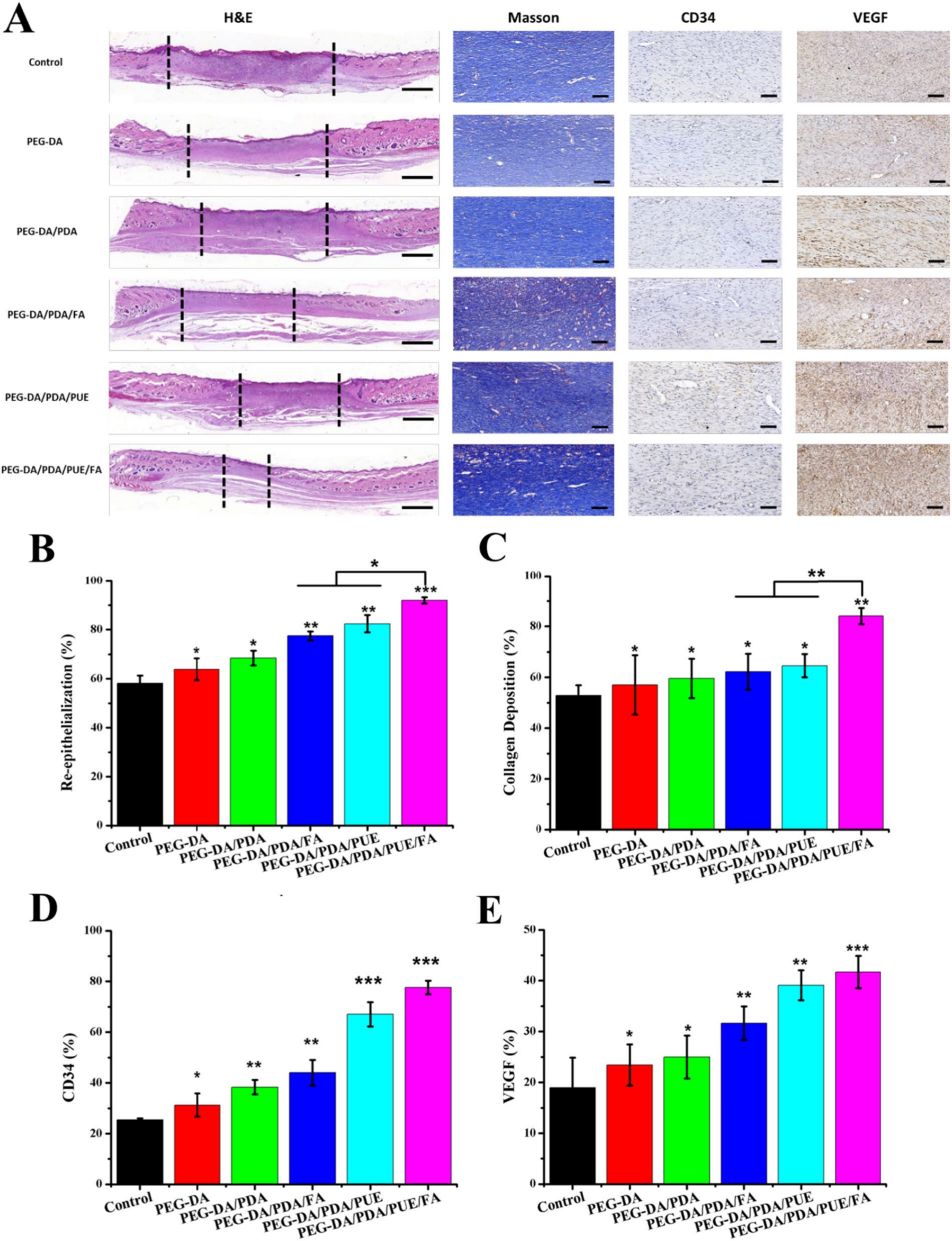
Histological analysis of wound healing. H&E, Masson’s trichrome, and immunohistochemical staining of CD34 and VEGF in the wound area (**A**; Scale bar in H&E is 1000 μm, Scale bar in masson, CD34 and VEGF staining is 200 μm). Semi-quantitative analysis of re-epithelization (**B**), collagen deposition (**C**), and the expression of CD34 (**D**) and VEGF protein (**E**) in the wound sections of control and hydrogel-treated rats at 15 days after surgery. ^*^P < 0.05, ^**^P < 0.01, and ^***^P < 0.001 when compared with the control group

Wound healing involves several biological processes, including hemostasis, migration, proliferation, and remodeling. After treatment for 15 days, histopathological changes occurred in different skin samples. H&E staining revealed that the wound healed in the PEG-DA/PDA/PUE/FA hydrogel group is faster than other groups (Fig. 7A and B). Additionally, the PEG-DA/PDA/PUE/FA hydrogel-treated wounds showed distinct recovery, which possessed mature fibrous tissues, well-organized fibroblasts, and blood capillaries (Fig. 7A). Therefore, the lack of inflammation and pathological abnormalities confirmed the histocompatibility of the PEG-DA/PDA/PUE/FA hydrogel. Collagen fibers are produced by fibroblasts, and the remodeling of these fibers is necessary during wound healing [44, 45]. Masson staining showed that the PEG-DA/PDA/PUE/FA hydrogels promoted the formation of collagen fibers (Fig. 7A and C). Moreover, the PEG-DA/PDA/PUE/FA hydrogels effectively upregulated the expression of CD34 protein, resulting in improved platelet-endothelial cell adhesion (Fig. 7A and D). VEGF had the highest expression in PEG-DA/PDA/PUE/FA hydrogel-treated wounds (Fig. 7A and E), which suggests that the vessel formation in PEG-DA/PDA/PUE/FA hydrogel is more than other groups.

The results of histological studies on wound healing showed that the PEG-DA/PDA/PUE/FA hydrogel dressings had antioxidant potential to promote wound healing. Moreover, the semipermeable nature and free radical-scavenging property of the PEG-DA/PDA/PUE/FA hydrogel wound dressings may be responsible for the early contraction of the wound and formation of fibrous tissue. These results demonstrate that the PEG-DA/PDA/PUE/FA hydrogel wound dressings can be used as candidate materials for wound applications such as repair and regeneration of damaged skin.

## Conclusion

In this study, a triple antioxidant nanocomposite hydrogel wound dressing is developed by incorporating PUE and FA into the dressings via PDA NPs, which maintains the drugs in the gel network for a long time. The hydrogel possesses excellent mechanical and anti-oxidant properties, which is beneficial for wound dressing. Taking advantage of three natural drugs for alleviating oxidative stress, the triple PEG-DA/PDA/PUE/FA hydrogel shows great potential for clinical application.

## Supplementary Information

**Additional file 1.** Additional table and figures.
