# Supplementary material for: More natural more better: triple natural anti-oxidant puerarin/ferulic acid/polydopamine incorporated hydrogel for wound healing
Source: J Nanobiotechnology. 2021 Aug 11;19:237. doi: 10.1186/s12951-021-00973-7 (PMC8359571; doi:10.1186/s12951-021-00973-7)
Supplement: Supplementary file 1 — Additional file 1. Additional table and figures. [file 12951_2021_973_MOESM1_ESM.docx]

**Additional file 1: Table S1. The nanostructured drug delivery systems for wound healing.**

| **Drug Delivery System** |  | **Substance** | **General Description** | **Drug** | **advantages** | **Ref** |
| --- | --- | --- | --- | --- | --- | --- |
| **Micelles** |  | **Poly(l-lysine-b-phenylalanine)**  **Poly (glutamic acid-b-phenylalanine)** | **Spontaneous self-assembly of copolymers** | **Curcumin and amphotericin B** | **1. Excellent biocompatibility,**  **2. Enhance wound contraction, granulation, and re-epithelialization**  **3. Mitigate inflammatory response.** | [1-2] |
| **Polymeric nanoparticles** |  | **Dopamine** | **Polymerized from natural, synthetic, and semi-synthetic polymers, with size in the range of 10-1000 nm** | **Tetracycline hydrochloride** | **1. Drug controlled/sustained release,**  **2. Biocompatibility,**  **3. Improve wound healing** | [3-4] |
| **Liposomes** |  | **Polyethylene glycol liposomes** | **Spherical and self-enclosed structures formed by one or more concentric lipid bilayers** | **Glucocorticoids** | **1. Alleviate local inflammation**  **2. Accelerate wound healing**  **3. Promote the formation of collagen matrix** | [5-6] |
| **Inorganic nanoparticles** |  | **Gold nanoparticles** | **Inorganic nanoparticles were polymerized from inorganic compound.**  **Such as quantum dots, magnetic NPs, and gold NPs.** | **Epigallocatechin gallate, and α-lipoic acid** | **1. Anti-inflammatory function**  **2. Antioxidative effect**  **3. Accelerate wound healing** | [7-8] |


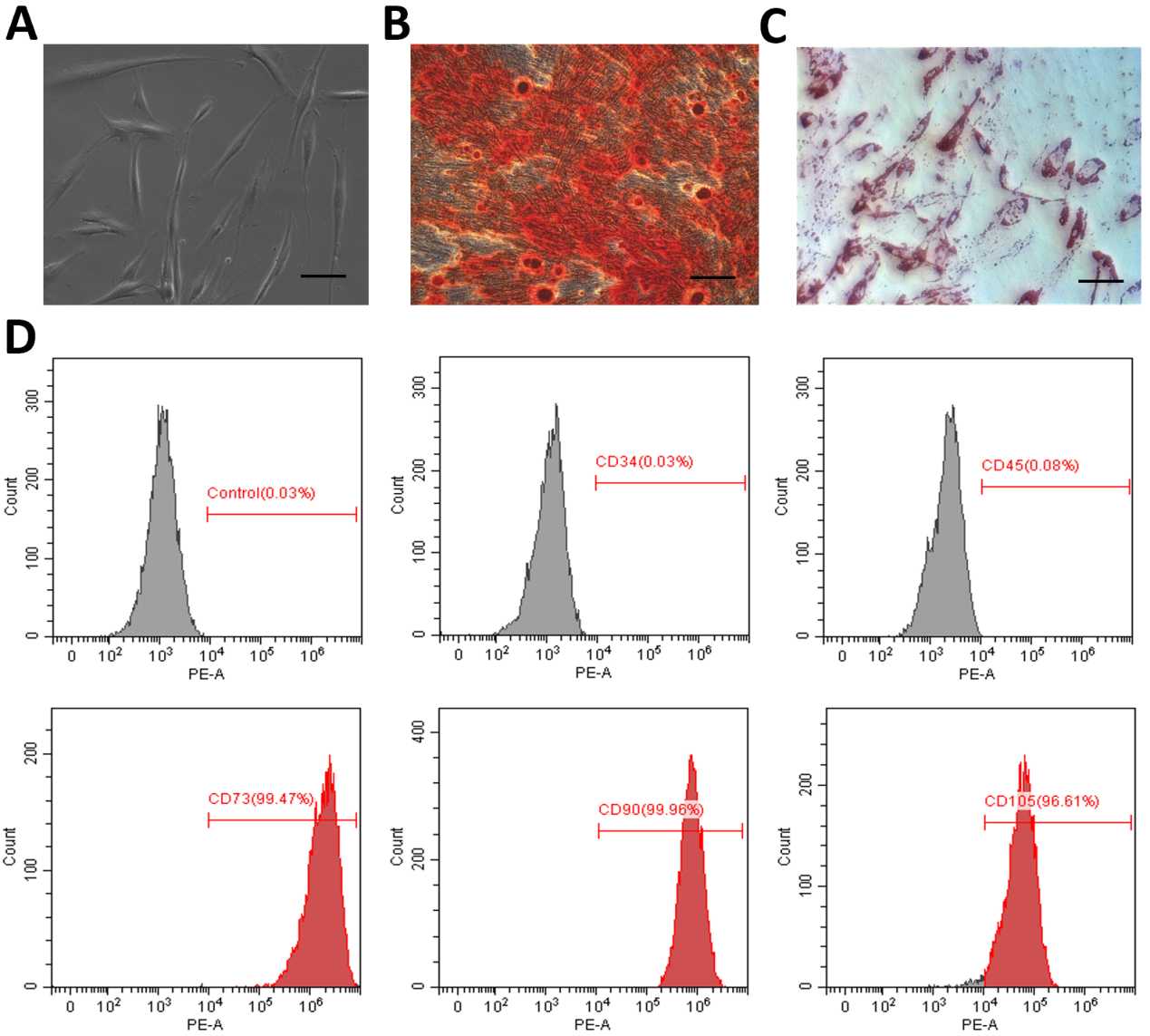


**Additional file 1: Figure S1. Human periodontal ligament stem cells (hPDLSCs) characterization.**

HPDLSCs showed the fibroblast-like morphology (A; Scale bar is 100 μm), osteogenic differentiation (B; Scale bar is 100 μm) and adipogenic differentiation (C; Scale bar is 100 μm). Flow cytometry showed that hPDLSCs negatively expressed CD34 and CD45, while positively expressed CD73, CD90 and CD105 (D).


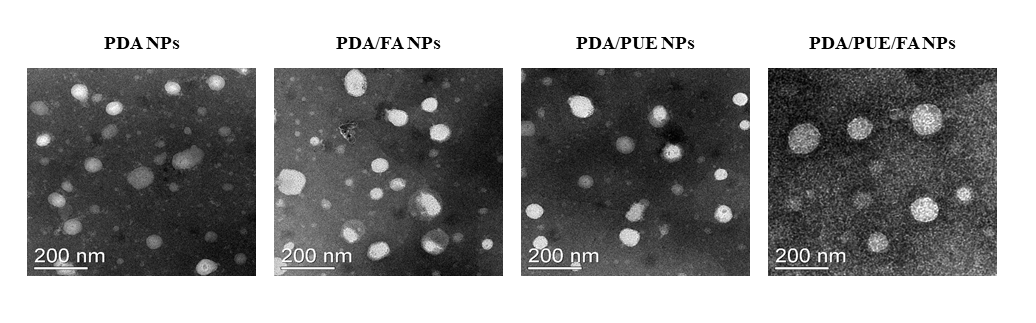


**Additional file 1: Figure S2. The morphological characterization of nanoparticles.**

TEM images of PDA NPs, PDA/FA NPs, PDA/PUE NPs, and PDA/PUE/FA NPs.


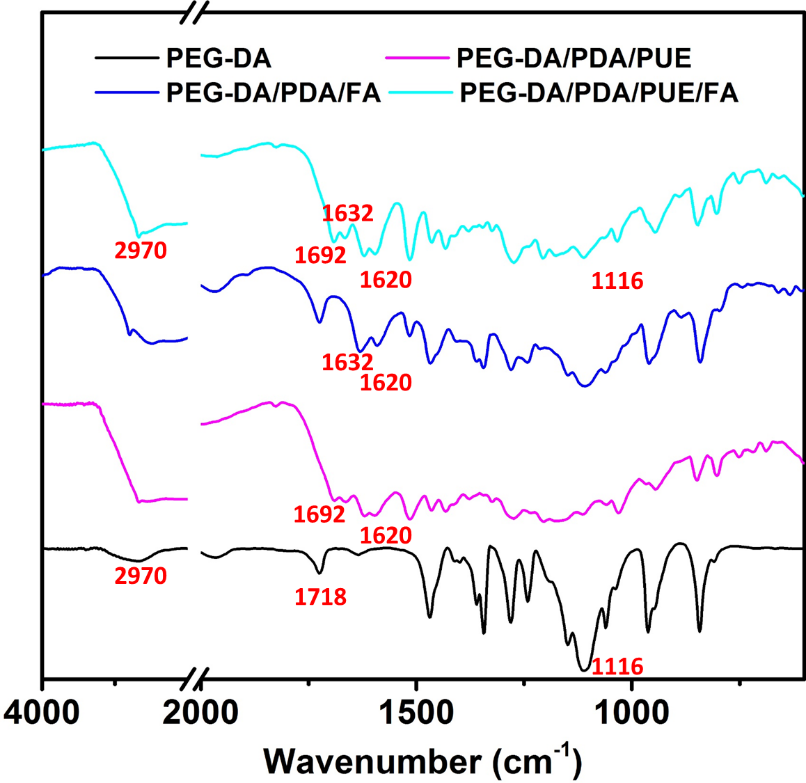


**Additional file 1: Figure S3. The FT-IR spectrum of the hydrogel.**

The FT-IR spectrum of PEG-DA, PEG-DA/PDA/PUE, PEG-DA/PDA/FA and PEG-DA/PDA/PUE/FA.

**Additional References:**

1. M Patel, T Nakaji-Hirabayashi, K Matsumura. Effect of dual-drug-releasing micelle-hydrogel composite on wound healing in vivo in full-thickness excision wound rat model. J BIOMED MATER RES A. 2019;107(5):1094-1106.

2. J Varshosaz, S Taymouri, M Minaiyan, F Rastegarnasab, A Baradaran. Development and in vitro/in vivo evaluation of HPMC/chitosan gel containing simvastatin loaded self-assembled nanomicelles as a potent wound healing agent. DRUG DEV IND PHARM. 2018;44(2):276-288.

3. Y Liu, Y Sui, C Liu, C Liu, M Wu, B Li, et al. A physically crosslinked polydopamine/nanocellulose hydrogel as potential versatile vehicles for drug delivery and wound healing. Carbohydr Polym. 2018;188:27-36.

4. JM Mofazzal, ZP Sahandi, BS Moosavi, ZK Sahandi, A Ghamarypour, AR Aref, et al. Nanomedicine and advanced technologies for burns: Preventing infection and facilitating wound healing. Adv Drug Deliv Rev. 2018;123:33-64.

5. A Gauthier, A Fisch, K Seuwen, B Baumgarten, H Ruffner, A Aebi, et al. Glucocorticoid-loaded liposomes induce a pro-resolution phenotype in human primary macrophages to support chronic wound healing. BIOMATERIALS. 2018;178:481-495.

6. R Cheng, L Liu, Y Xiang, Y Lu, L Deng, H Zhang, et al. Advanced liposome-loaded scaffolds for therapeutic and tissue engineering applications. BIOMATERIALS. 2020;232:119706.

7. SK Nethi, S Das, CR Patra, S Mukherjee. Recent advances in inorganic nanomaterials for wound-healing applications. Biomater Sci.2019;7(7):2652-2674.

8. JG Leu, SA Chen, HM Chen, WM Wu, CF Hung, YD Yao, et al. The effects of gold nanoparticles in wound healing with antioxidant epigallocatechin gallate and alpha-lipoic acid. NANOMEDICINE-UK. 2012;8(5):767-75.
